# Significant Detectivity Enhancement of Broad Spectral Organic–Inorganic Hybrid Photodiodes by C_60_ Film as Hole-Blocking Layer

**DOI:** 10.1186/s11671-021-03651-7

**Published:** 2022-01-25

**Authors:** Zhuoli Zhou, Guangmeng Liao, Xinyu Song, Qinyong Dai, Lei Sun, Yingquan Peng, Peng Wang

**Affiliations:** 1 College of Optical and Electronic Technology, China Jiliang University, Hangzhou, 310018 China; 2 Institute of Microelectronics, School of Physical Science and Technology, Lanzhou University, Lanzhou, 730000 China

**Keywords:** Broad spectral photodiodes, Organic–inorganic hybrid photodiodes, Planar-bulk heterojunction, Hole-blocking layer

## Abstract

As an important classification of photodetectors, broad spectral photodiodes are ubiquitous in the fields of industry and scientific research. Here, we reported a type of broad spectral organic–inorganic hybrid photodiodes (OIHPDs) based on planar-bulk heterojunction, which composed of 3,4,9,10-perylenetertracarboxylic dianhydride (PTCDA), copper phthalocyanine (CuPc) and fullerene (C_60_). In our research, the dark current of the OIHPD with 10 nm C_60_ film (10 nm-C_60_ OIHPD) was as low as 25.6 μA, which is about 63 times smaller than the dark current of the OIHPD without C_60_ film (C_60_-free OIHPD). It is considered that the significantly enhanced performance of 10 nm-C_60_ OIHPD is attributed to the introduction of the C_60_ film, which act as hole-blocking layer to reduce the dark current. And through the schematic energy level model combined with experimental measurements, the reason for the dark current change was well explained. Furthermore, the specific detectivity of 10 nm-C_60_ OIHPD was almost one order of magnitude larger than it of C_60_-free OIHPD, and a notable enhancement of over 10^11^ cm Hz^1/2^/W was obtained due to the fiercely reduced dark current. These results provide insights on how to improve the performance of organic photodiodes.

## Introduction

In optical information transmission and processing, photodetectors, which convert optical signals into electrical signals, are critical devices, especially in optical fiber communication [1], night vision [2], infrared remote sensing [3], imaging [4, 5], biomedical [6] and spectrometer [7]. At present, conventional inorganic photodetectors based on Gallium nitride (GaN), silicon (Si) and indium gallium arsenide (InGaAs) were well developed for detecting different sub-bands within the ultraviolet to near infrared range [8–10]. However, the flexibility of inorganic semiconductor materials is not satisfactory. Furthermore, some photodetectors based on inorganic semiconductor materials must be cooled during operation [11], which greatly restrict its further development and practical application. Thus, organic semiconductor materials have attracted widespread attention from researchers due to their good compatibility and special physical properties [12–15]. The development of organic–inorganic hybrid structures and the optimization of multicomponent organic heterojunctions are always good ideas for achieving broad spectral response through the complementarity of absorption spectra between different materials [16, 17]. For example, Yang et al. fabricated an organic photodetector with broad spectral photo-response from 200 to 1000 nm by adopting thick polymer bulk heterojunction composed of 1, 1-bis ((di-4-tolylamino) phenyl) cyclohexane (TAPC) and C_70_, and achieved an external quantum efficiency (*EQE*) over 1000%, a specific detectivity (D*) over 10^11^ Jones (cm Hz^1/2^/W) [18]. Wang et al. reported a mixed tin–lead perovskites (MASn_1−*x*_Pb_*x*_I_3_) photodetectors by using low-bandgap (FASnI_3_)_0.6_(MAPbI_3_)_0.4_ perovskite (FA = formamidinium) as the active layer, and achieved an *EQE* larger than 65% under − 0.2 V bias, a D* of 10^11^ ~ 10^12^ Jones in the wavelength range of 350–900 nm [19]. Han et al. developed a polymer photodetector by inserting cathode and anode interlayers, and achieved a D* over 10^12^ Jones in the wavelength range of 300–1700 nm [20]. Ma et al. utilized a ZnO/In_2_O_3_ heterojunction to improve sensing performance [21]. Zhang et al. fabricated a asymmetric supercapacitors with high energy density by using 3D hierarchical CoWO_4_/Co3O_4_ nanowire arrays [22]. And core–shell heterostructures were also used in nano structures [23]. Recently, our group has also demonstrated some broad spectral organic photodetectors based on planar heterojunction or hybrid planar-bulk heterojunction [24–26]. In addition, organic photodetectors have the advantages of low cost, high flexibility and large-area scalability, which make organic photodetectors have special research value and broad application prospects in the traditional optoelectronic field [27–30].

Generally speaking, the broad spectral photodetectors can be classified into three categories: phototransistors, photodiodes and photoconductors, and the structures include planar structure, bulk heterostructure and hybrid structure [31–33]. With further research, a series of methods have been adopted to improve the performance of organic photodetectors, such as developing new materials, optimizing device structure, doping quantum dots and inserting the inducing layers [34–38]. In this paper, we reported the broad spectral organic–inorganic hybrid photodiodes (OIHPDs) based on planar-bulk heterojunction of Si/C_60_/3,4,9,10-perylenetertracarboxylic dianhydride: copper phthalocyanine (PTCDA:CuPc) /Au. C_60_ presents numerous exciting chemical and physical properties and has been widely employed as an efficient trapping material in various optoelectronic applications. It is worth mentioning that the C_60_ film was introduced as a hole-blocking layer to enhance the barrier height for blocking hole transport. Thereby, the dark current was significantly reduced, and the spectral response was covered from visible light to near-infrared using PTCDA:CuPc as a light absorbing layer. The resulting organic photodetectors showed a specific detectivity over 10^11^ Jones in the spectral range of 405–655 nm.

## Methods

### Fabrication of Devices

**Fig. 1:**
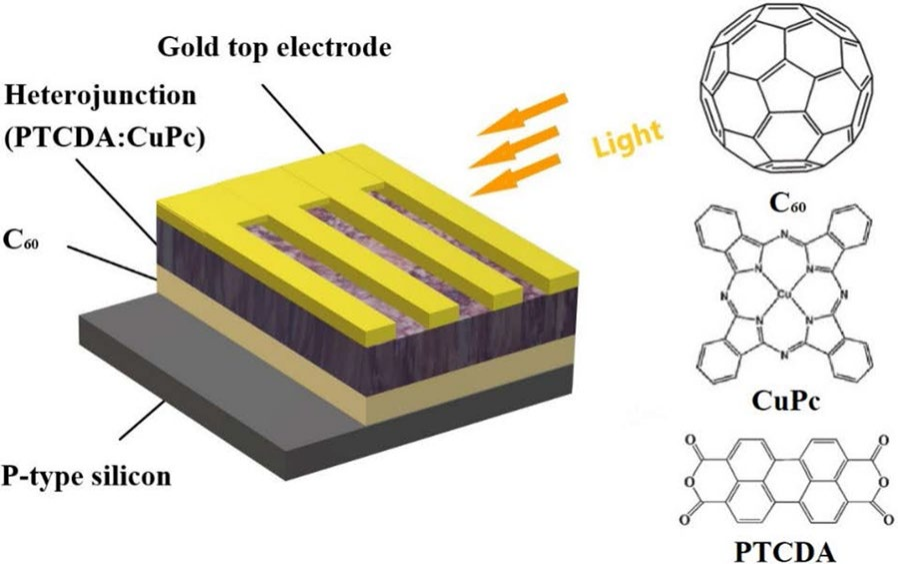
The schematic structure of devices based on hybrid planar-bulk heterojunction. The insets are molecular structures of PTCDA, CuPc and C_60_

The device configuration of the OIHPDs is depicted in Fig. 1, in which P-type silicon was used as the substrates, C_60_ films as buffer layers, organic bulk heterojunction as photosensitive layers and gold films as the top electrodes. Furthermore, the molecular structures of the organic materials are also inserted into Fig. 1 to understand the principle of the devices. For device fabrication, the p-type silicon substrates were successively cleaned by acetone, alcohol and deionized water for 10 min each, and then dried with floating N_2_ gas and baked in a vacuum oven at 60 °C for 20 min. Through a quartz crystal oscillator, it can monitor the different thickness of the films, and control the baffle and shadow masks, different thicknesses C_60_ (*δ* = 0, 5, 10, 20 and 30 nm, *δ* is the C_60_ thickness) films were deposited on the cleaned p-type silicon substrates by vacuum thermal evaporation. Following that, 30 nm-thick PTCDA:CuPc (weight ratio 1: 1, both purchased from Aladdin Biochemical Technology Co., Ltd.) bulk heterojunction films were vacuum evaporated on the top of C_60_ films. Next, the gold top electrodes were deposited on the organic films by shadow masks. During the deposition process, the chamber pressure was maintained below 3 × 10^–4^ Pa and the evaporation rate was kept at 0.1–0.2 Å/s. The effective area of each photodetector is 0.06 cm^2^.

### Characterization of Devices

The absorption spectra of films were measured by using TU-1901 spectrometer. All measurements were performed using a semiconductor characterization system in a dark chamber at room temperature. Laser diodes with wavelengths of 405 nm, 450 nm, 532 nm, 655 nm and 808 nm were used as the light source, and the different optical powers were realized by using neutral density filters.

## Results and Discussion

**Fig. 2:**
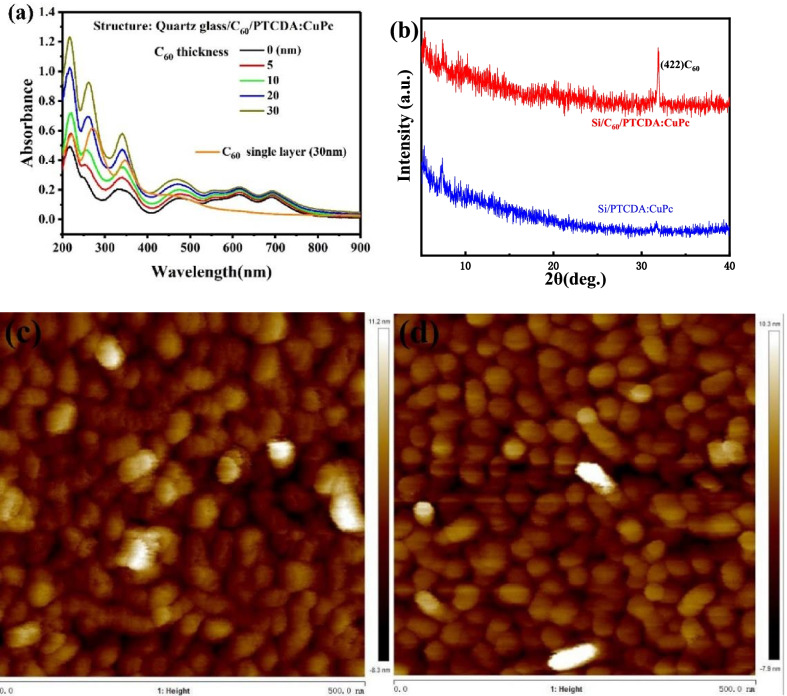
**a** Optical absorption spectra of C_60_ single layer (30 nm) and PTCDA:CuPc (30 nm) films deposited on C_60_ film of different thicknesses. All films were deposited on quartz glasses. **b** XRD spectrum of PTCDA:CuPc films with and without C_60_ film. And AFM images of **c** C_60_/PTCDA:CuPc and **d** PTCDA:CuPc films

Figure 2a depict the optical absorption spectra of 30 nm-thick PTCDA:CuPc films on different thicknesses C_60_ films and C_60_ single layer. Here, the PTCDA:CuPc films show strong light absorption in the spectrum range of 200–400 nm and a uniform light absorption in the spectrum range of 450–700 nm. It is worth noting that a remarkably enhanced absorption was found from 200 to 500 nm when different thickness C_60_ films were inserted between the interface of the quartz glasses and PTCDA:CuPc films, which could be attributed to the absorption of C_60_ film dominates in the spectrum range of 200–500 nm. Figure 2b–d shows the XRD and AFM images of PTCDA:CuPc on 10 nm C_60_ film and single PTCDA:CuPc film. The double layer show an additional peak at 31.9°. As shown in the AFM images, the surface of the PTCDA:CuPc film is similar with or without the C_60_ layer. However, the roughness of the photosensitive film on the C_60_ layer is slightly higher. This shows that the addition of C_60_ does not significantly improve the performance of the device optically, or even not at all. Therefore, we believe that the improvement of device performance comes from the hole blocking effect of C_60_.

The crystallite size (*D*) can be calculated by

1 $$D = k\lambda /(\beta \cos \theta ),$$

where *k* is a constant, equal to 0.89, *λ* is X-ray wavelength of 0.15405 nm, *β* is full width at half maxima (FWHM) of diffraction peak, *θ* is diffraction angle. The FWHM of the peak at 2*θ* = 31.9 is 0.1617°. The calculated crystallite size is about 50 nm, which is consistent with the AFM image.

**Fig. 3:**
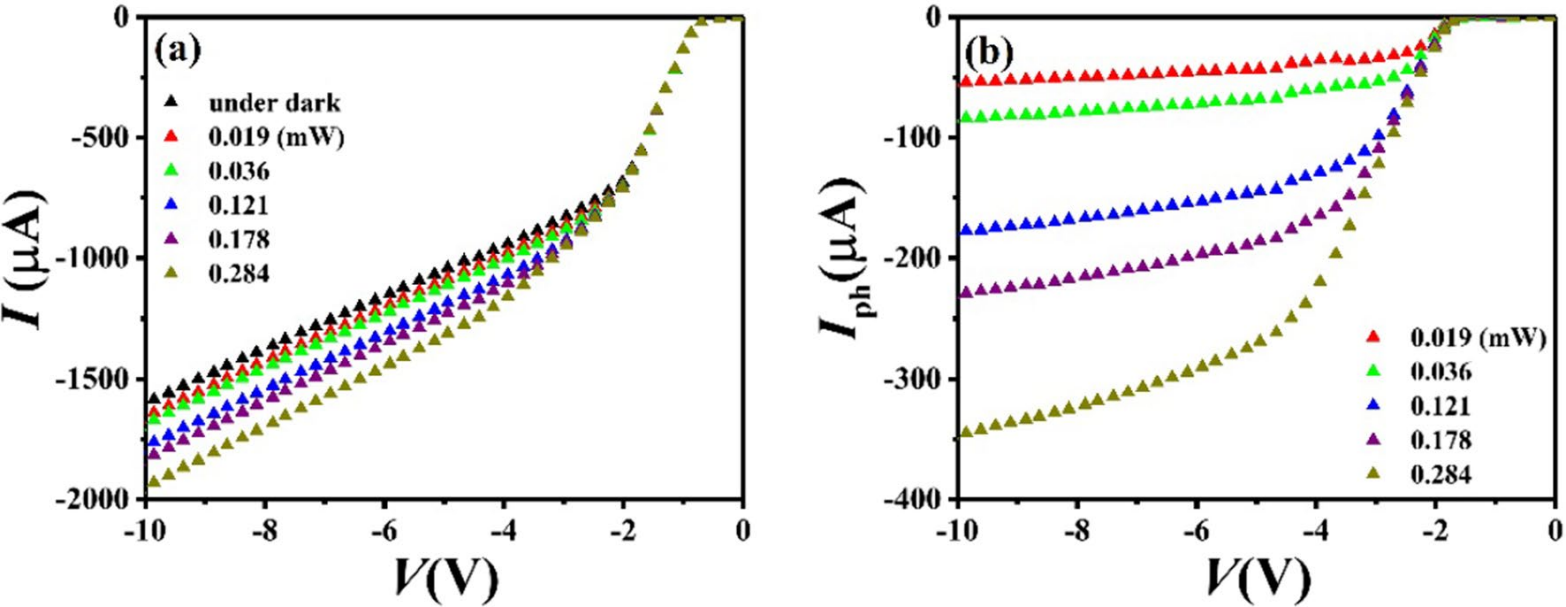
**a** The *I*–*V* characteristics of the device Si/PTCDA:CuPc (30 nm)/Au in the dark and under 532 nm laser illumination with different optical power. **b** The dependences of photocurrent on the reverse bias voltage under 532 nm laser illumination with different optical power

Figure 3a shows the typical *I*–*V* characteristics of the OIHPDs based on bulk heterojunction without C_60_ in the dark and under illumination of 532 nm laser. It is clear that the dark current is a bit large at a reverse bias voltage of − 10 V, which is as high as 1615.9 μA. The dependences of photocurrent on the reverse bias voltage are shown in Fig. 3b. It is seen that the photocurrent increases obviously with the incident optical power and gradually becomes saturated with reverse bias voltage increasing, which exhibits standard photodiode characteristics.

As an important parameter of photodetectors, the photoresponsivity (*R*) is defined as the value of the photocurrent (*I*_ph_) which generated in the external circuit under illumination of unit incident optical power (*P*_in_), that is [39]

2 $$\text{R} = \frac{{\text{I}}_{\text{ph}}}{{\text{P}}_{\text{in}}}.$$

Another important parameter characterizing photodetectors is the specific detectivity (D*). Here we ignore the power spectral density of other noise sources such as flicker or thermal noise, it can be described as [40]

3 $${\text{D}}^{*}=\frac{\text{R}}{\sqrt{{2}{\text{q}}{\text{J}}_{\text{dark}}}},$$

where *q* is the elementary electric charge, *J*_dark_ is the dark current density.

**Fig. 4:**
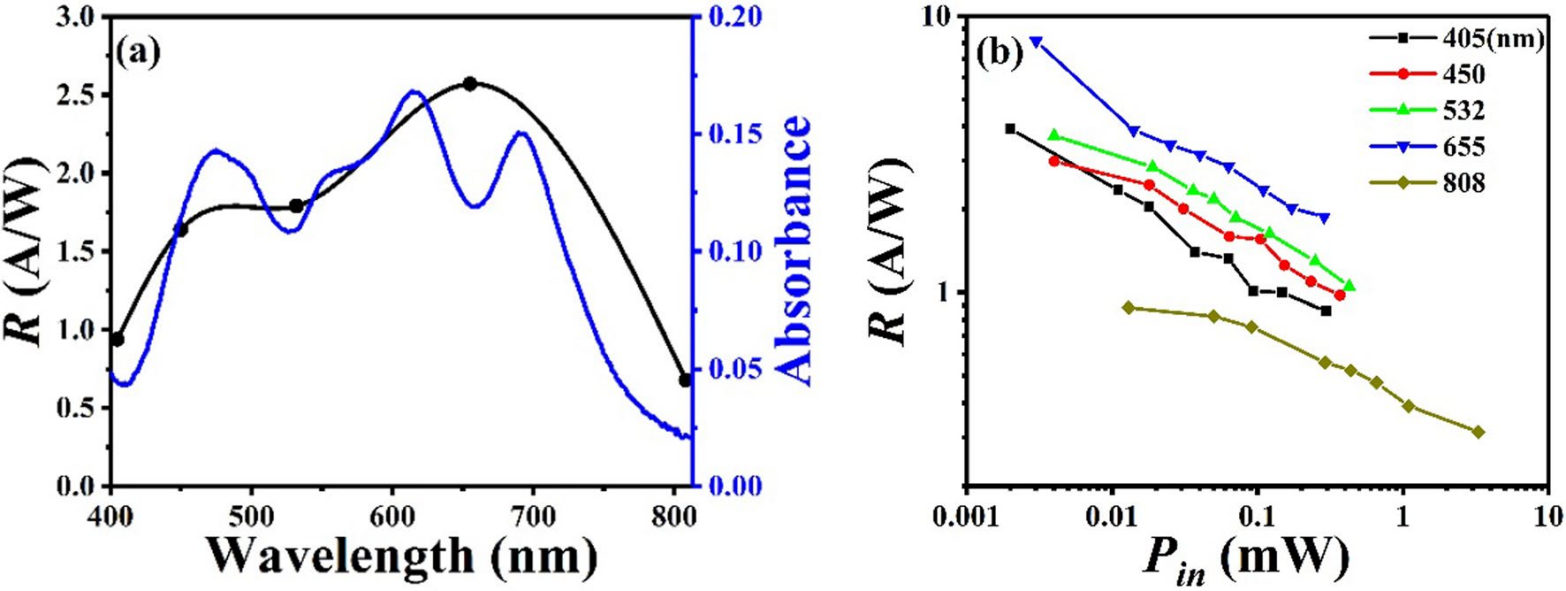
**a** The dependence of photoresponsivity *R* on the wavelength. **b** The logarithmic dependences of *R* on the incident optical power *P*_in_. All results were obtained at a reverse bias voltage of − 10 V and an incident optical power of ~ 0.1 mW

Figure 4a shows the dependence of photoresponsivity *R* on the wavelength at a reverse bias voltage of − 10 V and an incident optical power of ~ 0.1 mW. In the broad spectral response range from 405 to 808 nm, the responsivities of the OIHPD without C_60_ film (C_60_-free OIHPD) are all greater than 0.68 A/W. Especially, at the wavelength of 655 nm, the highest responsivity value is as high as 2.57 A/W. In addition, photoresponsivity generally follows the absorption spectrum of heterojunction films. As shown in Fig. 4b, under different wavelengths of light, the photoresponsivity decreased linearly with the incident light power increased in the logarithmic coordinate. Moreover, the maximal photoresponsivity is 8.13 A/W under 655 nm wavelength illumination with an incident optical power of 0.003 mW, and the minimal photoresponsivity is 0.32 A/W under 808 nm wavelength illumination with an incident optical power of 3.29 mW.

**Fig. 5:**
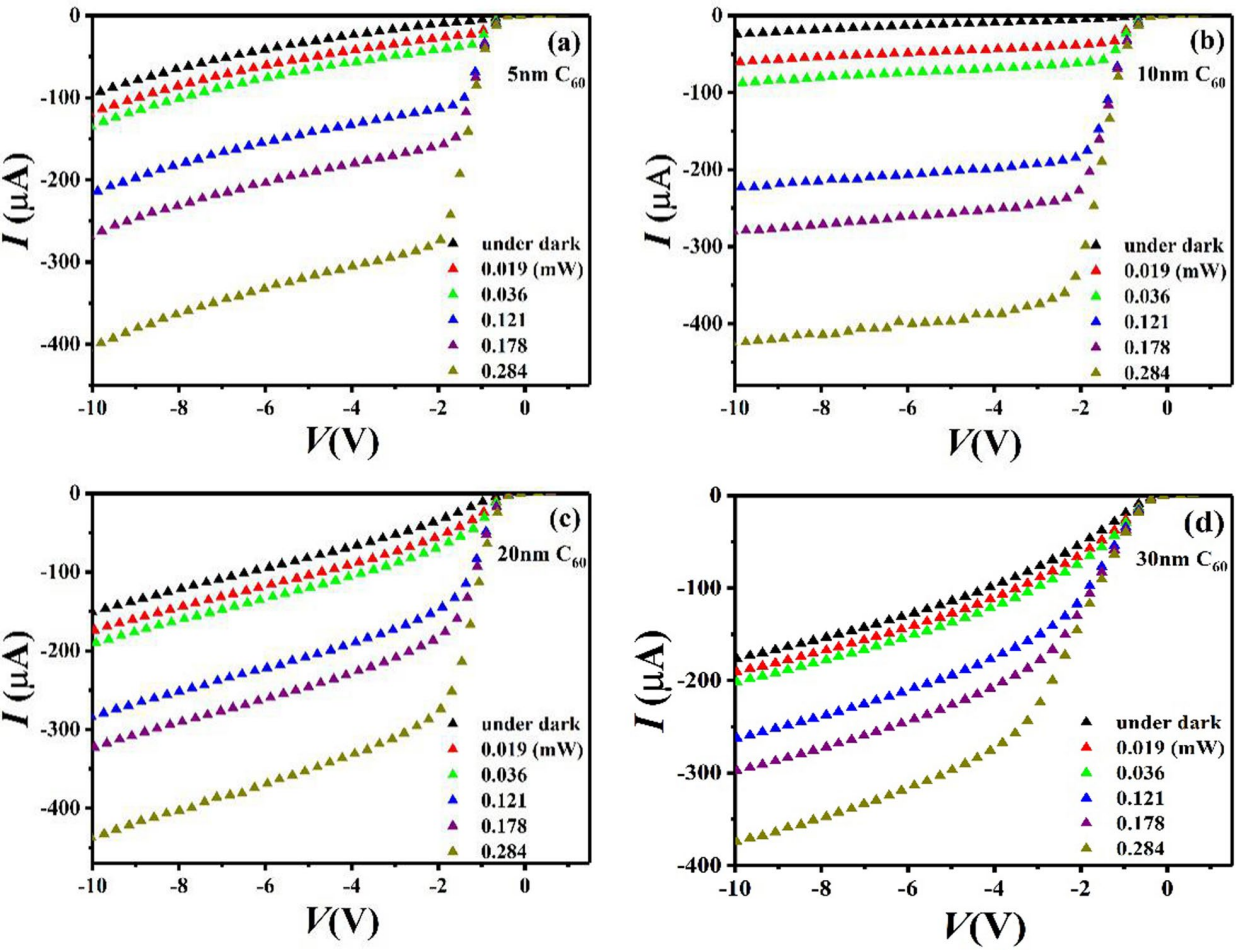
The *I*–*V* characteristics of the device Si/C_60_/PTCDA:CuPc (30 nm)/Au in the dark and under 532 nm laser illumination with different optical power. The thicknesses of C_60_ films are **a** 5 nm, **b** 10 nm, **c** 20 nm and **d** 30 nm, respectively

**Fig. 6:**
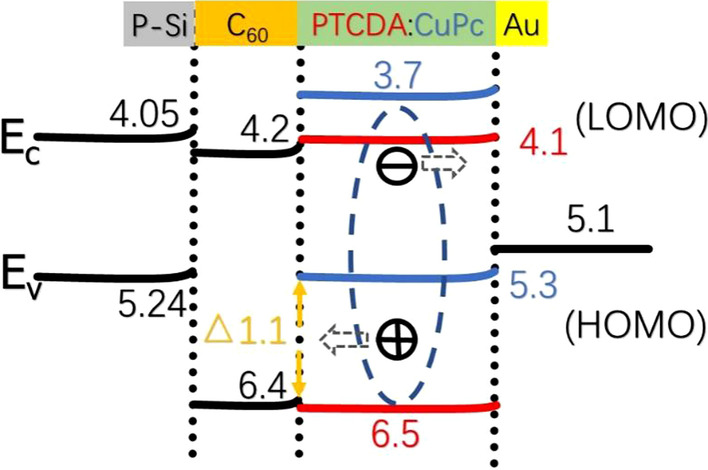
The energy level diagram and charge generation kinetics and transport mechanisms

Figure 5 shows the *I*–*V* characteristics of the OIHPDs with different thicknesses C_60_ films (C_60_-OIHPDs) in the dark and under 532 nm laser illumination. All measurements were conducted in the same reverse bias voltage range with different incident optical power. It is observed that the dark currents were 92.1 μA (Fig. 5a), 25.6 μA (Fig. 5b), 149.0 μA (Fig. 5c) and 179.8 μA (Fig. 5d), which corresponded to the C_60_ thickness of 5 nm, 10 nm, 20 nm, 30 nm, respectively. Additionally, the C_60_-OIHPDs exhibited much lower dark current (Fig. 3a vs Fig. 5), and the minimum dark current of C_60_-OIHPDs was 25.6 μA when the C_60_ film thickness is 10 nm, which is about 63 times smaller than it of the C_60_-free OIHPD. Moreover, it is interesting to find that the dark current decreased first and then increased with increasing C_60_ thickness. Considering the energy level, a mechanism explanation as shown in Fig. 6 is proposed to support the phenomenon of the above OIHPDs. It is noteworthy that the energy levels of valence band (*E*_v_) (5.24 eV) of p-type silicon, the highest occupied molecular orbital (HOMO) (5.3 eV) of CuPc and the work function of the gold electrode (5.1 eV) match well for hole transport in the absence of the C_60_ layer. Therefore, the holes can reach p-type silicon easily under a very low voltage bias, which causes a large dark current in the C_60_-free OIHPD. Fortunately, the HOMO (6.4 eV) of C_60_ is higher than the HOMO (5.3 eV) of CuPc, which means it will match better (*Δ* = 1.1 eV) for blocking hole transport. This is consistent with the experimental results that a high dark current of 1615.9 μA in the C_60_-free OIHPD and a low current of 25.6 μA in the C_60_-OIHPD. However, as an excellent electron transport material with high electron mobility (> 1.3 cm^2^ V^−1^ s^−1^) [41], a small amount of electrons can still easily reach the lowest occupied molecular orbital LUMO (4.2 eV) of C_60_ from the guide band *E*_c_ (4.05 eV) of p-type silicon. Thus, as the thickness of C_60_ film increasing, we infer that the injection of electrons is enhanced. In this regard, it has been reported that the strain relaxation in the multilayer film is related to the film thickness [42, 43]. Consequently, it is reasonable to assume that a thicker C_60_ film can provide broader space for the multilayer film, thereby more effectively alleviating the interplanar stress. In addition, the promotion of electron transport by the C_60_ film has become the dominant factor affecting dark current, which may be due to the increase of the dark current when the thickness of the C_60_ film exceeds 10 nm.

**Fig. 7:**
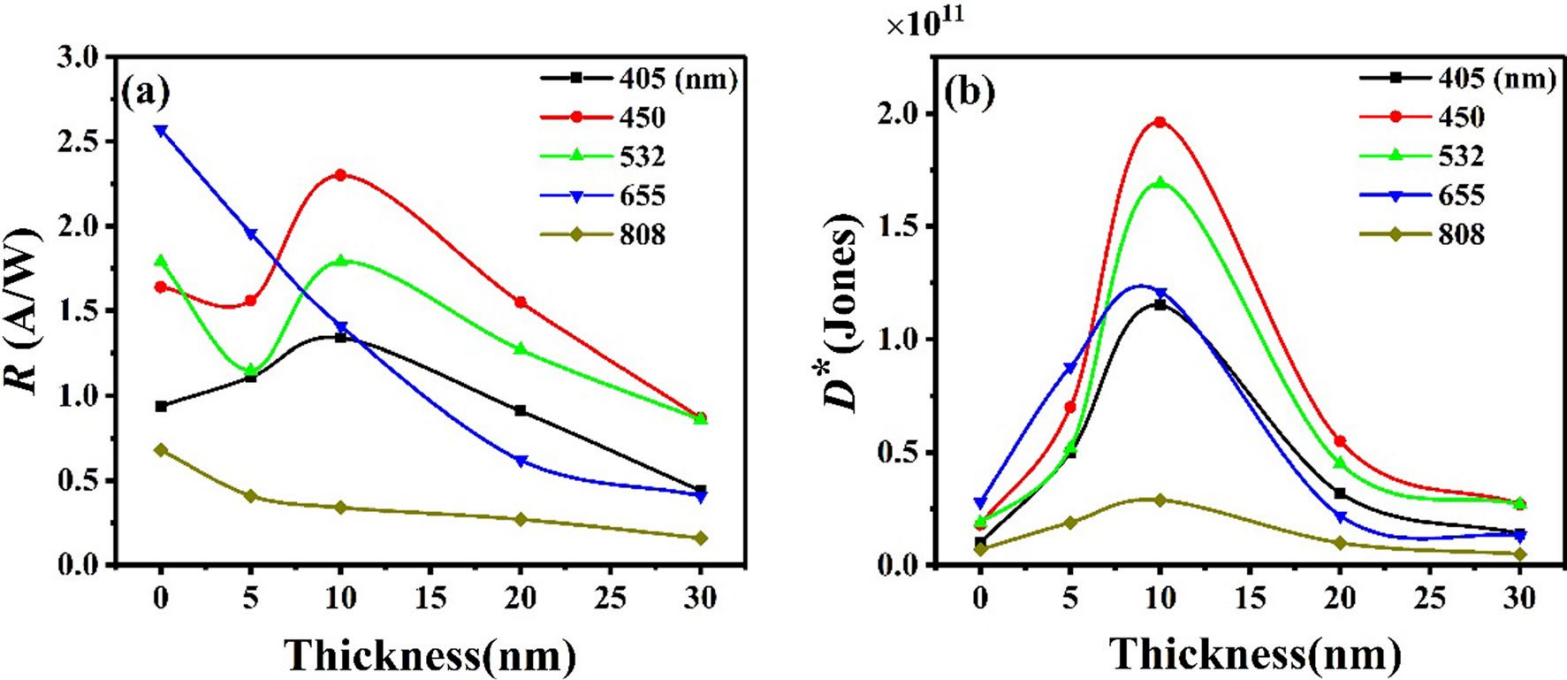
The dependences of **a** photoresponsivity *R* on the different C_60_ thicknesses, **b** specific detectivity D* on the thickness for different wavelengths. All results were obtained at a reverse bias voltage of − 10 V and an incident optical power of ~ 0.1 mW

In order to better understand the effects of C_60_ thickness on device performance, the relationship curves about the dependences of photoresponsivity *R* on the thickness of C_60_ films is plotted in Fig. 7a. Under the wavelength of 405–532 nm, there is an overall trend that *R* firstly increased and then decreased with C_60_ thickness increasing, and the maximum value of *R* was reached with 10 nm thick C_60_ film. It is indicated that C_60_ film does enhance optional absorption from 200 to 500 nm, which is consistent with the absorption spectrum of C_60_ single layer (Fig. 2). But the thicker C_60_ film will also lead to smaller photocurrent and smaller *R*, this is probably because C_60_ film hinders the transmission of portion of the photogenerated carriers. As for the wavelength of 450–532 nm, *R* significantly decreased when the thickness of C_60_ increasing from 0 to 5 nm, we speculate that the inhibitory effect of the C_60_ film on the photocurrent is greater than the enhancement effect at this time. Under the wavelength of 655–808 nm, *R* decreased continuously as the thickness of C_60_ increasing, since the C_60_ film has no significant enhancement effect at the wavelength of 655–808 nm. Figure 7b shows the dependences of D* on the thickness for different wavelengths. The D* of device under different wavelengths illumination firstly increased with C_60_ thickness increasing and reach a maximum value at 10 nm, then decreased. The results above manifest that the optimized C_60_ thickness is 10 nm, which makes the D* reach the maximum. For the OIHPD with 10 nm C_60_ film (10 nm-C_60_ OIHPD), the D* over 10^11^ Jones in the wavelength range of 405–655 nm, and the highest D* value is 1.96 × 10^11^ under the illumination of 450 nm wavelength. Compared with C_60_-free OIHPD, the D* of 10 nm-C_60_ OIHPD is almost one order of magnitude larger in the wavelength range of 405–808 nm (details in Table 1).

**Table 1:** Comparison of the photosensitive performance of broad spectral OIHPDs with different C_60_ thicknesses

Device											
C_60_ thickness	*R* (AW^−1^)^a^					D* (× 10^11^ Jones)^a^					*I*_dark_ (μA)^a^
Wavelength (nm)	405	450	532	655	808	405	450	532	655	808	
0-nm	0.94	1.64	1.79	2.57	0.68	0.10	0.18	0.19	0.28	0.07	1615.0
5-nm	1.11	1.56	1.15	1.96	0.41	0.50	0.70	0.52	0.88	0.19	92.1
10-nm	1.34	2.30	1.97	1.41	0.34	1.15	1.96	1.69	1.21	0.29	25.6
20-nm	0.91	1.55	1.27	0.62	0.27	0.32	0.55	0.45	0.22	0.10	149.0
30-nm	0.44	0.87	0.86	0.41	0.16	0.14	0.28	0.28	0.13	0.05	179.8

^a^All results were obtained at a reverse bias voltage of − 10 V and an incident optical power of ~ 0.1 mW

**Fig. 8:**
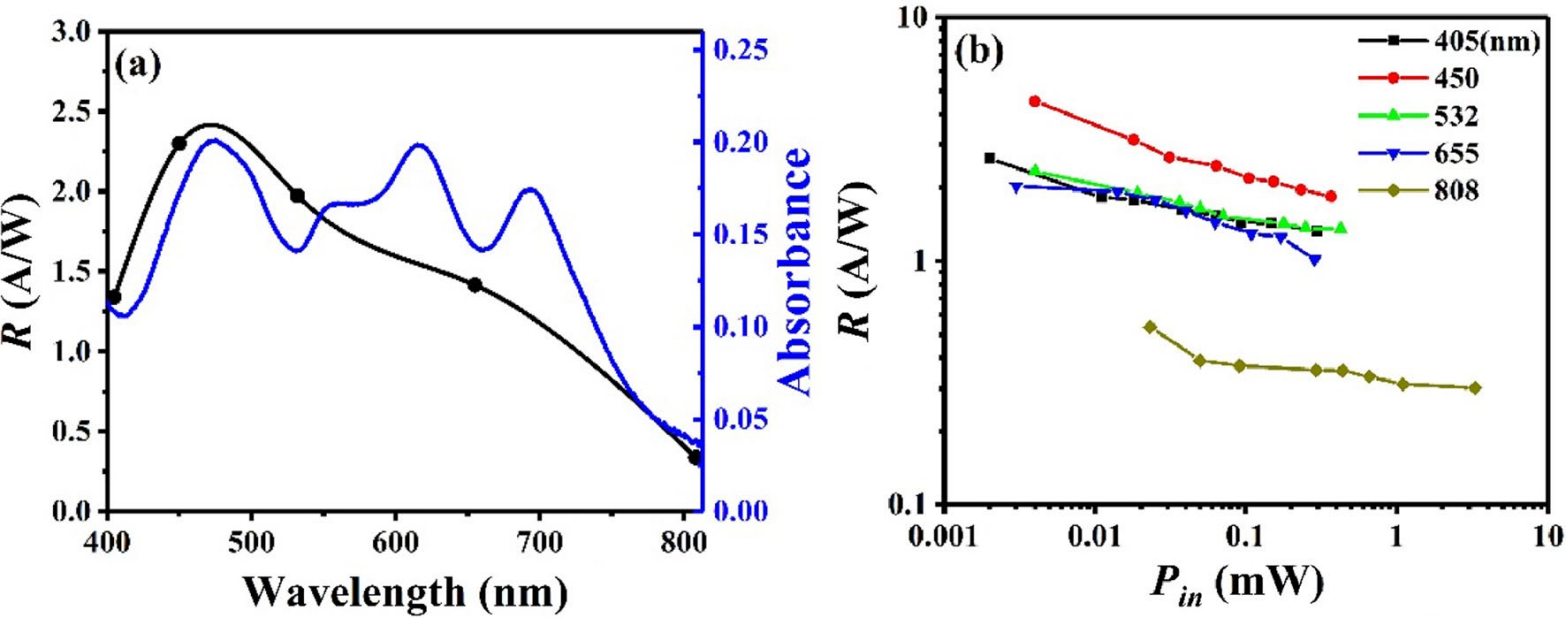
**a** The dependence of photoresponsivity *R* on the wavelength. **b** The logarithmic dependences of *R* on the incident optical power *P*_in_. All results were obtained at a reverse bias voltage of − 10 V and an incident optical power of ~ 0.1 mW

Figure 8a shows the dependence of photoresponsivity *R* on the wavelength for an incident optical power of ~ 0.1 mW at − 10 V. It is worth mentioning that the peak value of responsivity of 10 nm-C_60_ OIHPD changes to around 450 nm from 655 nm of C_60_-free OIHPD (Fig. 8a vs Fig. 4a) due to the addition of C_60_ film, which enhanced the absorption spectrum from 200 and 500 nm (Fig. 2). Furthermore, significantly reduced responsivity values are obtained at the wavelength of 655 nm and 808 nm (Fig. 8a vs Fig. 4a) due to C_60_ film which hinders the transmission of portion of the photo-generated carriers. As shown in Fig. 8b, it is seen that the 10 nm-C_60_ OIHPD has the best responsivity under the illumination of 450 nm because of the influence of C_60_ film. And the maximal responsivity is 4.53 A/W at an incident optical power of 0.004 mW of 450 nm, the minimal responsivity is 0.30 A/W at an incident optical power of 3.29 mW of 808 nm, respectively.

## Conclusion

In summary, the photodiodes based on hybrid planar-bulk heterojunction with different thicknesses C_60_ films were fabricated and characterized. The broad spectral region response from visible to near-infrared demonstrated that using C_60_ films as hole-blocking layer can effectively enhance the performance of broad spectral OIHPDs. Specifically, the OIHPD with 10 nm-C_60_ film exhibited the optimized performance with a much lower dark current of 25.6 μA, which is about 63 times smaller than that of C_60_-free OIHPD. A schematic energy level model combined with experimental measurements is well capable of explaining the origin of decreased dark current. Furthermore, the D* of the 10 nm-C_60_ OIHPD was almost one order of magnitude larger than the C_60_-free photodiode, and a notable enhancement of over 10^11^ Jones was obtained due to the fiercely reduced dark current.

## Data Availability

The data in the manuscript were obtained from our measurements, so we will not share the data.

## Abbreviations

OIHPD: Organic–inorganic hybrid photodiode
PTCDA: 3,4,9,10-Perylenetertracarboxylic dianhydride
CuPc: Copper phthalocyanine
C_60_: Fullerene
TAPC: 1,1-Bis ((di-4-tolylamino) phenyl) cyclohexane
*EQE*: External quantum efficiency
*D**: Specific detectivity
*R*: Photoresponsivity
XRD: Diffraction of x-rays
FWHM: Full width at half maxima
AFM: Atomic force microscope
